# Precursors of Prenatal Attachment and Anxiety during Pregnancy in Women Who Procreate Naturally and Pregnant Women following Assisted Reproduction Technology

**DOI:** 10.3390/ijerph20206945

**Published:** 2023-10-19

**Authors:** Monica Pellerone, Juan Martinez-Torvisco, Stesy Giuseppa Razza, Elena Commodari, Sandra Miccichè

**Affiliations:** 1 Faculty of Human and Social Sciences, Kore University of Enna, 94100 Enna, Italy; stesygiuseppa.razza@unikorestudent.it (S.G.R.); micciche.sandra@gmail.com (S.M.); 2 Department of Cognitive, Social and Organizational Psychology, La Laguna University, 38200 Santa Cruz de Tenerife, Spain; jtorvisc@ull.edu.es; 3 Department of Educational Sciences, University of Catania, 95126 Catania, Italy; ecommoda@unict.it

**Keywords:** pregnancy, prenatal attachment, anxiety, infertility, ART

## Abstract

The process of adaptation of the woman to pregnancy seems to be influenced by many factors, such as the type of conception, the mother’s age, the possible presence of other children, and socio-cultural factors. Women who conceived with an assisted reproductive technique are emotionally vulnerable; compared with pregnant women who procreated naturally, they manifest elevated anxiety, which seems to be correlated to the fright of being separated from their child. Objectives of the present research are as follows: (1) to analyze the relationship between age, gestational age, time expectancy, previous failed attempts, perception of a high-risk pregnancy, and presence of other children, with the level of maternal–fetal attachment (MFA); (2) explore the level of maternal–infant attachment and anxiety by comparing the control and experimental group; (3) to measure a possible relationship between anxiety levels and MFA in ART pregnant women; (4) to identify variables predictive of prenatal attachment. The study group is formed by ninety-five women aged between 18–42 years (M = 30.57; S.D. = 5.47), pregnant from the 23rd to the 37th week (M = 28.95; S.D. = 3.99); on which 50 women who procreate naturally and 45 pregnant women following assisted reproductive technology. They completed: Maternal–Fetal Attachment Scale (MFAS), State–Trait Anxiety Inventory (STAI), and ad hoc questionnaire to collect anamnestic data. The results show the presence of a correlation between gestational age and waiting period, between the number of assisted fertilization attempts with the worry about their ability to become pregnant, but a negative correlation between pregnancy weeks and the level of maternal–fetal attachment. The study shows the predictive role of anxiety on the MFA. The applications and indications for future research are analyzed.

## 1. Introduction

Pregnancy is one of the most intense and complex experiences for a woman. It represents a period of significant change, full of uncertainties, frustrations, and at times, disappointments [1,2]. It determines a particular psychic organization, which develops in a woman in order to adequately prepare for the birth of her child [3].

Contrary to early studies [4], which described pregnancy as a period of relative well-being and “immunity” from psychiatric disorders, more recent studies instead, show that some women might manifest the first symptoms of depression during pregnancy; instead, for other women, previous episodes of mood disorders represent a risk factor for recurrence or aggravation of prenatal symptoms [5].

One of the main predisposing factors to the manifestation of mental disorders is the condition of infertility. It has repercussions on the psychological well-being not only of the individual but of the parental couple [6].

Its discovery introduces a crisis event that is often associated with the path of medically assisted procreation (MAP), a further source of stress due to invasive and painful medical procedures, and required economic and time investment [7,8]. In other words, the emotional load posed by medical tests and the path leading to the diagnosis follows the initial stress and discomfort, caused by the discovery of infertility.

In fact, the literature has shown that women of infertile couples suffer from anxiety and/or depressive symptoms to a greater extent than women whose conception occurs naturally [9,10]; these symptoms may influence optimal maternal–infant attachment [11].

The idea of the existence of a mother–fetus attachment is not a recent development in the psychology of pregnancy; Deutsch [12] was the first to hypothesize that attachment could begin during gestation. In this phase, the mother and fetus live in symbiosis: the mother communicates to the fetus everything she perceives; she thinks about and imagines her child; their relationship is regulated not only emotionally but also nutritionally [13].

Winnicott [14], introducing the concept of primary maternal concern, highlighted how this relationship originates before birth, in the affective commitment that the parental mind develops towards the expected child.

Most studies on prenatal attachment indicate that this bond increases progressively as pregnancy progresses, starting from the second trimester of gestation with the perception of the first fetal movements [15,16]. In particular, during the fifth month of pregnancy, the fetal movements become clearly perceptible by the mother: this is much more decisive than the ultrasound participation regarding the emotional work that the woman performs in modulating communication towards the fetus.

The adaptation process of the pregnant woman in these moments is very important: the woman takes note of a living and autonomous presence in her body, causing a reorganization of her own internal relationships to prepare for the separation, that is required with the event of the birth [17,18].

Up to this moment, mother and child have been a unit; from now on, the presence of another being is felt, determining the possibility of a first form of attachment that arises between mother and fetus. In fact, the literature underlines that an elevated maternal–fetus attachment is correlated to a positive parent–child relationship in the post-natal phase, which could contribute to structuring a secure and functional attachment [19].

Therefore, the formation of a healthy maternal representation of the unborn child and the emerging recognition of the fetus as an individual separate from herself would represent significant mediating variables between prenatal attachment and parent–child relation in the post-natal phase [20].

## 2. Prenatal Attachment and Maternal–Fetal Representation during Vitro Fertilization Path

The interest in the literature on prenatal attachment and maternal–infant representations of pregnant women who procreate through assisted fertilization is relatively recent and limited.

The literature shows how women’s reactions to pregnancy, after an experience of infertility, tend to fall between two extremities. On the one hand, denial and avoidance (denial/rejection): there are women who deny pregnancy, do not follow medical prescriptions, and are unable to provide appropriate prenatal care for several months. On the other hand, there may be a hyper-vigilant attitude: a state of exaggerated fear is highlighted for every minimal and insignificant symptom; they need constant reassurance about the evolution of the pregnancy.

Research shows that in women who conceive naturally and in those undergoing assisted reproduction, the level of anxiety appears similar in the first and second trimesters, although anxiety tends to increase in pregnant women undergoing medical-assisted procreation (MAP) during the third trimester [21].

Elevated levels of anxiety during pregnancy appear to be related to the concern for the survival and health of the child, possible harm to the child during delivery, and the mother’s fear of being separated from the newborn [22,23].

The story of an infertile woman is often the path of a woman who has suffered many major losses in her life [24]; when she finds out she is pregnant she tends to create mental representations and fantasies about a child who will be perfect or about being a special mother [25].

In some circumstances, these women experience and describe pregnancy only in terms of a gratifying and fulfilling experience, while denying the psychological and physical difficulties of this state. They appear less interested in documenting the birth of the child and their parenting experience, and they are less inclined to participate in birth preparation courses. They communicate little with the unborn child and delay all preparations for the birth of the baby. They control every aspect of their pregnancy in an effort to distance themselves from the normal feelings of unease and ambivalence [26,27,28].

The literature shows that during a medically assisted pregnancy, multiple aspects emerge, such as: fear of a possible pregnancy termination, fear of conceiving an “unhealthy” child, anxiety about having a high-risk pregnancy, and ambiguity due to feeling neither infertile nor pregnant [29,30]. To these factors we can also add a sense of ambiguity in the link with a fetus (conceived in a laboratory), excessive anxiety felt about one’s ability to manage pregnancy, childbirth, and parenting duties, as well as fear that others will find out how conception occurred [31]. It can be hypothesized that the pregnant women who conceive with assisted conception techniques—due to the long periods of infertility they have suffered—may show an elevated level of affective emotional involvement during pregnancy, affecting the quality of maternal–fetal attachment [32].

The predictors of MFA could be divided into three categories: (a) pregnancy-related dimensions; (b) demographic variables; and (c) psychosocial characteristics. In reference to the first dimension, the literature has underlined that in women who have conceived through assisted fertilization methods, prenatal attachment begins with pregnancy [33], and increases with pregnancy progression simultaneously with anxiety levels [32,33,34,35]. The mother–infant attachment appears to increase before the second trimester prenatal screening testing, and after receiving the results at fourteen weeks of gestational age [35]. In particular, couples who conceived with ART seem to manifest a higher prenatal attachment than couples who conceived spontaneously [36]. A subsequent study found that women who conceived after ART had less adjustment to the self and the well-being of the baby, but more ability to adjust in the process of preparing for childbirth and in fulfilling the role of mother [37].

The greater gestational age also seems to be associated with a higher level of MFA [38], probably due to more intense fetal activity and more frequent fetal monitoring. In confirming data, a study by Pellerone and Micciche [39] shows that mother–infant attachment is influenced not only by gestational age but also by the waiting time of pregnancy and by the number of failed attempts in women who conceive with ART treatment. In detail, the study underlined that the women who became pregnant spontaneously tend to manifest a higher level of differentiation from the fetus than IVF pregnant women. Furthermore, gestational age appeared to be a predictive variable of the ability to perceive themselves as independent from the fetus they carried in their womb.

In reference to demographic predictors, in the study carried out by McMahon [22], the advanced age of mothers who conceive naturally was associated with a low level of maternal–infant attachment. Whereas in ART mothers, younger women—starting at the gestational age of thirty-six weeks—tend to manifest an elevated level of maternal–fetus attachment.

Furthermore, the literature agrees that in women who conceive naturally, with increasing age, there is a reduction in the level of anxiety, thanks to the acquisition of problem-solving abilities and stress management related to pregnancy. However, in women who conceive by fertilization methods, due to the advanced age resulting in high-risk pregnancy, there is an increase in anxiety levels, above all in mothers over 35 years of age [40].

In reference to the psychological dimensions, the literature has evaluated the mood of pregnant women in correlation with depression, anxiety, and prenatal attachment. A part of the research underlined the absence of a correlation between attachment and the general psychological state of pregnant women, in particular the absence of anxious or depressive symptomatology [32,33,39].

Other studies have shown that anxiety and depression can have a negative impact on prenatal attachment [33,41,42]. The literature shows conflicting data: for example, in the study carried out by McMahon and colleagues [23], pregnant women who conceived with ART, manifested better levels of prenatal attachment but mostly pregnancy-related state anxiety. Similarly, a study conducted in the United Kingdom has noted, although on a medium level, a negative correlation between anxiety and prenatal attachment levels, in the group of women who conceive with the in vitro fertilization method [43].

On the contrary, other studies [33,39] found that state anxiety in ART women was not a predictive variable of MFA. Likewise, Udry-Jørgensen et al. [35] underlined no difference in symptoms of anxiety and depression between ART and parents of spontaneous conceivers, although in both groups, as levels of anxiety and depression increased, their prenatal attachment decreased.

## 3. Research Goals and Hypothesis

The research is conducted by comparing an experimental group of women, who are receiving assisted reproduction treatment, with a control group made up of naturally pregnant women. Objectives of the present work are as follows: to analyze the relationship between independent variables (that is age, gestational age, time expectancy, previous failed attempts, perception of a high-risk pregnancy, and presence of other sons) with the level of MFA; to compare the maternal–fetus attachment (MFA) and anxiety level into women who conceive naturally and women who receive assisted reproductive treatment; finally, to identify variables predictive of MFA in both groups of pregnant women.

According to the literature, we hypothesized the presence of a correlation between age, gestational age, waiting time, perception of a high-risk pregnancy, the fear of not being pregnant, and maternal–fetus attachment in the experimental group.

Into the ART women group, we hypothesized the manifestation of a lower level of MFA but a higher level of anxiety, and the existence of a correlation between anxiety level and MFA, due to the long periods of infertility they have suffered.

We hypothesized the predictive role of anxiety, the fear of not being pregnant, the perinatal loss event, and the perception of a high-risk pregnancy on the prenatal attachment level in the experimental group.

Finally, we suppose the same results in the group of women who conceive with the ICSI technique, as it is considered the most effective but invasive methodology, due to the complexity and duration of the treatment.

## 4. Research Method

### 4.1. Participants and Procedures

A cross-sectional research design was used. The study was conducted between January and December 2020 in collaboration with the Family Counseling Service (implemented by the Local Health Authority) during the COVID-19 pandemic, which made it difficult to collect a large amount of data.

All pregnant women who turned to the public health service in order to attend the preparation course for childbirth were involved. We used the non-random convenience sampling method.

The participant group is formed by ninety-five pregnant women, aged between 18 and 42 years (M = 30.57; S.D. = 5.47), from the twenty-third to the thirty-seventh week (M = 28.95; S.D. = 3.99), of which:

(a) Fifty pregnant women (control group), who conceived naturally, selected between the twenty-fourth and the thirty-seventh pregnancy week (M = 31.20; S.D. = 5.10), aged between 18 and 41 years (M = 29.94; S.D. = 5.92);
(b) Forty-five pregnant women (experimental group) who were receiving an assisted reproduction treatment, aged between 21 and 42 years (M = 31.27; S.D. = 4.88), selected between the twenty-third to the thirty-seventh pregnancy week (M = 28.56; S.D. = 3.60).

Based on the self-assessment, pregnant women who indicated they wanted to participate in the study and met the study inclusion criteria were allowed access to the compilation tools.

The inclusion criteria were as follows: participants’ age between 18 and 45 years, gestational age between the 2nd and 3rd trimester, natural or assisted conception method.

Participation in the research was voluntary, there was no compensation, and anonymity was guaranteed to all participants. In order to guarantee the confidentiality of the research, the management of the tools was entrusted only to the research group. The research participants filled in the informed consent form.

### 4.2. Instruments

Both control and experimental groups completed: a constructed ad hoc questionnaire; the Maternal–Fetal Attachment Scale [44], in order to measure the maternal–fetal bonding; and the State–Trait Anxiety Inventory [45] for the assessment of the trait and state anxiety.

The ad hoc questionnaire measures: age, gestational age, type of fertilization technique used, waiting period, the perception of a possible high-risk pregnancy, the fear or worry about their ability to become pregnant, the number of assisted fertilization attempts, previous abortions, and presence of other children.

The Maternal–Fetal Attachment Scale (MFAS), an instrument aimed at measurement of prenatal attachment, consists of 24 items divided into five sub-scales:

(a) *Differentiation from Self* is represented by items showing the woman’s joy at thought of “another from herself” and the attribution of her child’s name (example: “I like to watch my belly move while the child is kicking”).
(b) *Interaction with the fetus*, that underlines the woman’s intention to talk to her fetus and refer to him/her by a nickname (example: “I talk about the child using nicknames o terms of Endearment”).
(c) *Attributing characteristics and intentions*, that highlights the woman’s recurring thoughts about what she may be feeling and process the fetus inside the own belly, or on the possible personality characteristics of fetus based on movements (example: “I can almost imagine what personality mine will have my child by how it moves”).
(d) *Giving of Self* is represented by actions that show the woman’s willingness to commit herself during pregnancy in order to prevent potential harm to the fetus (example: “I eat healthy so that my child can benefit from a healthy diet”).
(e) *Role Taking* is represented by items that highlight the woman’s ability to imagine oneself as a mother in the future and therefore thoughts related to maternal roles in feeding and caring for her child (example: “I imagine taking care of the child”).

In the present study, Cronbach’s alpha coefficient is equal to 0.69 in the control group and equal to 0.67 in the experimental group.

The State–Trait Anxiety Inventory consists of 40 items, which are divided into two sub-scales, the STAI T-Anxiety Scale (the trait anxiety), and the STAI-S Anxiety Scale (the state anxiety) on a four-point Likert scale point.

A. *State anxiety* represents a constellation of nervousness, fear, and discomfort induced by situations that are perceived as dangerous during a precise moment. Therefore, it is a measure of the level of contingent anxiety, i.e., related to feelings of insecurity and helplessness, which could lead to a consequent behavior of escape and avoidance from the dangerous situation.
B. *Trait anxiety* represents a constellation of feelings of stress and daily discomfort; therefore, it can be considered the tendency to perceive stressful situations as dangerous with a consequent tendency to respond to situations with varying intensity.

The Italian version used [46] presents a degree of internal consistency equal to 0.83. In the present study, Cronbach’s alpha coefficient is equal to 0.78 in the control group, and equal to 0.72 in the experimental group.

## 5. Data Analysis

In reference to the preliminary analysis, descriptive statistics were conducted in order to measure all independent variables in both groups of pregnant women.

To measure the correlation between all independent variables with MFA in both groups, two Pearson’s correlations were carried out.

In order to compare levels of MFA and anxiety in both groups, the Independent Samples T Test analysis was conducted.

Another Pearson’s correlation was carried out to measure the possible correlation between the anxiety level and maternal–fetal attachment in the experimental group.

An analysis of multiple mediations with two mediating variables forming a causal chain was carried out through PROCESS [47]. In detail, we assessed the relationship between MFA and anxiety through serial mediation with the fear of not being pregnant, the perinatal loss event, and the perception of a high-risk pregnancy. This analysis was performed on all experimental groups, and in women who conceived with the ICSI technique. A multiple mediation model is probably the most realistic model that allows X’s effect on Y to be transmitted through more than one mediator simultaneously. The mediation analysis allows for the evaluation of whether one independent variable (X) affects another dependent variable (Y) through a third variable (the mediating variable).

## 6. Preliminary Analysis

In reference to the preliminary analysis, Table 1 shows the descriptive statistics on the control group (Table 1).

Table 2 shows the descriptive statistics on the experimental group.

In reference to the fertilization technique, the descriptive analysis shows that 73.3% have used the ICSI (intracytoplasmic sperm injection), followed by 22.2% who have used the IVF (in vitro fertilization) and only 4.5% the IUI (intrauterine insemination). It seems interesting to underline that only a small part of the sample used intrauterine insemination, although it is considered the least invasive technique, as it consists of taking a sample of seminal fluid, prepared and optimized in the laboratory before being introduced into the uterus via a small catheter.

In the case of in vitro fertilization technique, sperm and oocytes are inserted into the culture liquid without any further human intervention.

Most of the sample underwent a procedure of intracytoplasmic sperm injection, considered the medically assisted procreation technique with the highest pregnancy rates, in which the best spermatozoa are isolated and injected into each available oocyte.

## 7. Results

In reference to the first research hypothesis, in the control group, the Pearson’s analysis underlines the presence of a correlation between gestational age with the fear of not being pregnant and the perception of a high-risk pregnancy. The presence of other sons (who were born from a previous natural or assisted pregnancy) seems to be correlated with age, gestational age, and the perception of a risk pregnancy (Table 3). The data seems to confirm the research hypothesis.

Confirming the research hypothesis, in the experimental group, Table 4 underlines that gestational age is correlated with the waiting period and the presence of other children, but negatively with the MFA level; the number of assisted fertilization attempts is related to fear about their ability to become pregnant.

In reference to the second hypothesis, an Independent Samples T Test analysis was carried out, in order to compare maternal–fetal attachment and the level of anxiety in both groups. Confirming the hypothesis, Table 5 shows significant differences in MFA total score and state anxiety level. The analysis of average scores underlines how the state anxiety appears to be higher in the experimental group, which also shows a lower maternal–infant attachment than the control group.

In reference to the third hypothesis, in the experimental group, Pearson’s correlation shows that: the “Role Taking” sub-dimension is correlated with the “Differentiation from Self” and “Interaction with Fetus” sub-dimensions. The general level of MFA is negatively correlated with “Interaction with fetus” and “Attributing characteristics and intentions” sub-dimensions. The analysis underlines the presence of a negative correlation between MFA and anxiety (both state and trait anxiety). The third hypothesis is confirmed (Table 6).

The fourth hypothesis of the study assessed the mediating role of “Fear of not being pregnant” (MV1) and “Perception of the high-risk pregnancy” (MV2) on the relationship between MFA (as an independent variable, IV) and anxiety (as a dependent variable, DV) within the experimental group.

To check this, an analysis of multiple mediations with two mediating parallel variables is carried out. One mediating variable called role mediated by fear of not being pregnant (MV1, fear in advance), and the second mediating variable named perception of a high-risk pregnancy (MV2, risk in advance) with MFA (IV) and anxiety (DV) are included through PROCESS v. 4.2 (Model 4) to test the hypothesis model.

The linear regression analysis shows, first, that MFA is not a significant predictor of “Fear of not being pregnant” (β = −0.137, S.E. = −0.003, t = −0.910 *p* < 0.367). MFA is also not found to be a significant predictor of “Perception of the high-risk pregnancy” (β = −0.001, S.E. = 0.004, t = −0.669, *p* < 0.669). Furthermore, the role mediated by the variable “Fear of not being pregnant” (MV1) shows a non-significant relationship on the state anxiety (β = 2.706, S.E. = 3.500, t = 0.773, *p* < 0.443); this is path b1. However, MV1 was found to have a significant impact on the trait anxiety (β = −7.543, S.E. = 2.691, t = −2.803, *p* < 0.007); this is path b2.

The total effect of MFA on anxiety (S) through mediating variables (MV1 and MV2) was found to be significant (β = −0.206, S.E = 0.075 t = −2.720, *p* < 0.009, LLCI = −0.358 and ULCI = −0.053). The direct effect of IV on DV shows a significant impact (β = −0.211, S.E = 0.071 t = −2.961, *p* < 0.005, LLCI = −0.356 and ULCI = −0.067).

Furthermore, we examined if the construct of MFA has an indirect effect through MV1 (fear) on the construct anxiety (Y), obtaining the following results.

(a) Indirect effect 1 (a1 ∗ b1): MFA →fear →anxiety; a1 (−0.0031) ∗ b1 (2.706) = −0.0084 (LLCI = −0.0356, ULCI = 0.0121). Because zero falls between the lower and upper bound of the 95% confidence interval, we infer that the indirect effect of MFA (X) through fear (MV1) on anxiety (Y) was not statistically significant.
(b) Indirect effect 2 (a2 ∗ b2): MFA → risk → anxiety; a2 (−0.0019) ∗ b2 (−7.543) = 0.0144 (LLCI = −0.0462, ULCI = 0.0887). Because zero (the null) falls between the lower and upper bound of the 95% confidence interval, we assume that the indirect effect of MFA (X) through risk (MV2) on anxiety (Y) was also not significantly different from zero.

The specific indirect effect contrast definition shows the construct fear minus risk (C1), effect = −0.0228 (LLCI = −0.1057, ULCI = 0.0423). Hence, neither of the two mediators used in the model mediated the relationship of MFA on anxiety.

Finally, we also tried to find out how the variable number of pregnancy attempts would act in a simple mediation model and the results also confirm the lack of relationship between VI and DV mediating the number of attempts. The indirect effect results show a β = 0.0017, (LLCI = −0.0318, ULCI = 0.0340). Because zero (the null) falls between the lower and upper bound of the 95% confidence interval, we suggest that the indirect effect of MFA (X) through the number of attempts (MV3) on anxiety (Y) was also not statistically significant.

**Figure 1:**
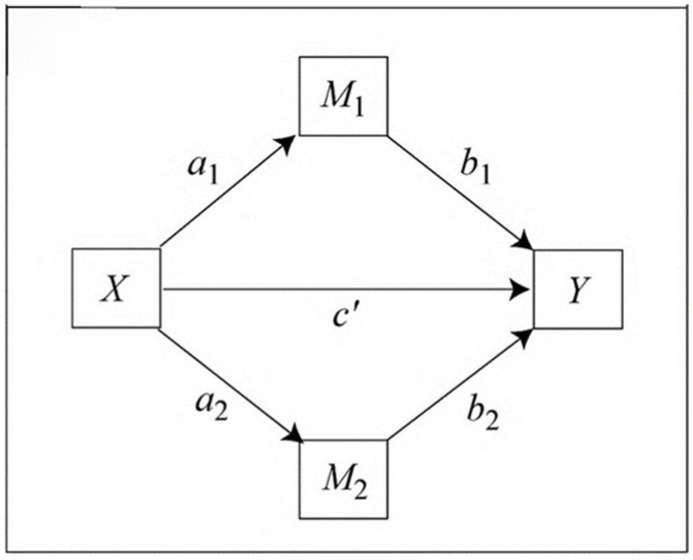
The parallel mediation model. Abbreviations: X = MFAS; Y = STAI; M_1_ = fear of not being pregnant; M_2_ = perception of the high-risk pregnancy.

**Figure 2:**
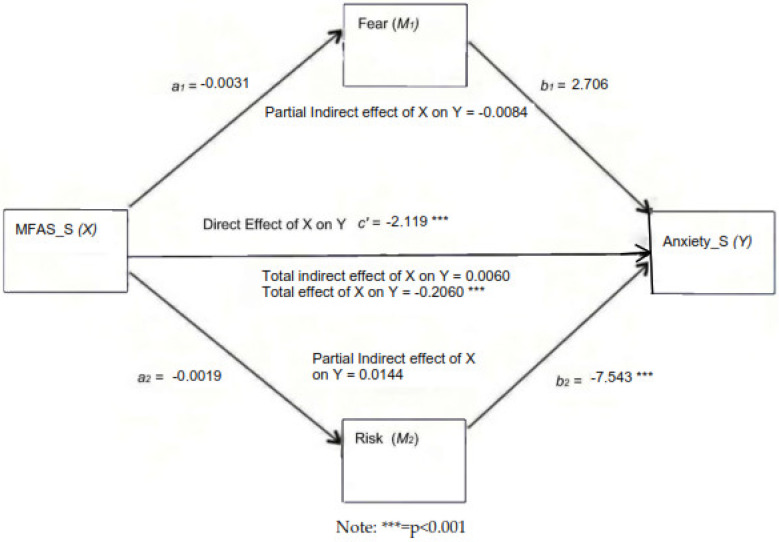
The precursor of prenatal attachment. Abbreviations: Fear = fear of not being pregnant; Risk = perception of the high-risk pregnancy.

The parallel mediation model summary is presented in Table 7. Figure 1 shows the theoretical design of the parallel mediation model and Figure 2 shows the coefficient values from the relationship between predicted, criteria and mediating variables.

Regarding the model summary total effect, it reveals a value of R-Sq of 0.1468, that is to say, that 14.68% of the variance is explained by the model (MSE = 80.809, F = 7.400, df2 = 43.00, *p* < 0.009).

The model analyzed shows a partial mediation since both total and direct effects are significant.

Also, indirect effects are not significant, and we suggest that there is no mediation relationship between the MFA (X) variable and the anxiety (Y) variable. Since the multiplication of the signs of the coefficients a_1_, b_1_, and c’ gives a positive result, we can highlight that the type of model is complementary.

The explanation for why the mediating variable may not be significant is due to the fact that these ones are not strongly enough related to the independent variable to be considered an effective mediator.

Finally, a complementary analysis was carried out to determine whether the type of technique helped to predict the multiple mediation model as in the previous analysis (Figure 2) with similar variables, that is: the mediating role of MV1 (fear) and MV2 (risk) on the relationship between MFA as independent variable (IV) and anxiety as dependent variable (DV) within experimental group, selecting only participants that have been used ICSI technique (used by 73.3% of the experimental sample).

The results reveal that firstly, the MFA is not a significant predictor (negative) on MV1 (β = −0.0024, S.E. = −0.0035, t = −0.6774 *p* = 0.5034).

MFA is also not found to be a significant predictor of MV2 (β = −0.0026, S.E. = 0.0056, t = −0.4674, *p* = 0.6435); MV1 (role mediated by fear of not being pregnant) shows a non-significant relationship on anxiety (state) (β = 3.0558, S.E. = 4.7874, t = 0.6383, *p* = 0.5283); this is path b1.

However, MV1 was found to have a significant impact on anxiety (S) (β = −7.8613, S.E. = 3.2872, t = −2.3915, *p* = 0.0235); this is path b2.

The total effect of MFA on anxiety (state) through mediating variables, MV1 and MV2 was found to be significant (β = −0.2061, S.E. = 0.0973 t = −2.1179, *p* = 0.0423, LLCI = −0.4045 and ULCI = −0.0076). The direct effect of IV on DV shows a significant impact (β = −0.2170, S.E. = 0.0926 t = −2.3430, *p* = 0.0262, LLCI = −0.4064 and ULCI = −0.0276).

**Figure 3:**
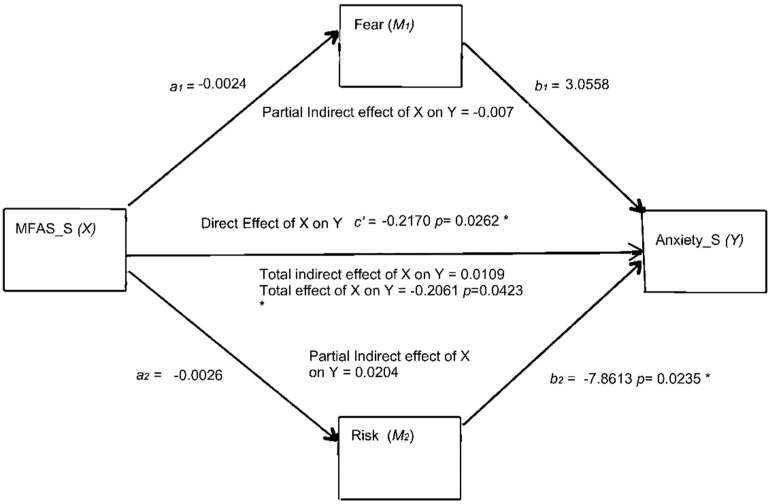
The precursor of prenatal attachment using ICSI fertilization technique. Abbreviations: Fear = fear of not being pregnant; Risk = perception of the high-risk pregnancy. *= *p* < 0.05.

Regarding the model summary total effect (Figure 3), it reveals a value of R-Sq of 0.1264, that is to say, that 12.64% of the variance is explained by the model (MSE = 94.335, F = 4.4853, df2 = 31.00, *p* = 0.423). The model analyzed shows a partial mediation since both total and direct effects are significant.

Also, indirect effects are not significant; we suggest that there is no mediation relationship between the MFA (X) variable and the anxiety (Y) variable when the fertilization technique ISCI was selected (n = 33 participants).

## 8. Discussion

The maturational process towards parenthood takes shape when a couple decides to become a family and tries to have a child, thus opening up mental space and emotional investment for the child they want. The imagined child, even if it has not yet been born, exists in the mind of the future parents; around his presence, the man and the woman construct mental representations, live emotions, build images about the future, and couple projects.

The present research was conducted in order to determine prenatal attachment precursors and anxiety levels of pregnant women, comparing women who procreate naturally and women who resort to artificial insemination.

According to the literature, the study assumes the presence of a relationship between age, pregnancy week, waiting time, the perception of a high-risk pregnancy, the fear of not being pregnant, previous abortion experiences, and maternal–fetal attachment.

Furthermore, the study assumed that ART pregnant women have reduced maternal–fetus bonding, but elevated state anxiety due to their expectations, and desire to have a child; this could be due to the long periods of infertility they have suffered and their investment in pregnancy. For this reason, the study also presupposed the presence of a relation between maternal–fetus attachment and anxiety in pregnant women using assisted reproductive technology.

Finally, we hypothesized the predictive role of maternal–infant attachment on the state anxiety, a role mediated by the fear of not being pregnant, other perinatal loss events, and the perception of a high-risk pregnancy in the experimental group.

In confirmation of the first hypothesis, in the control group, the correlation analysis shows the relationship between gestational age with the fear of not being pregnant and the perception of a high-risk pregnancy. A correlation between the presence of other sons with mother age, gestational age, and the perception of an unsafe pregnancy has been demonstrated.

Data underline that as the pregnancy progresses, the fear of not being able to bring the pregnancy to term and the perception of a high-risk pregnancy seem to increase. Otherwise, with increasing age and weeks of pregnancy, despite the presence of other sons, the perception of a risky pregnancy does not decrease but increases.

These data confirm how gestational age and the consequent perception of fetal movement are correlated with maternal–infant attachment [48,49]; in some pregnant women, the perception of fetal movements could lead to an increase in the fear of not carrying a pregnancy to term, and the perception of a risky pregnancy for oneself and for one’s child.

Differently, within the ART pregnant women, there is a correlation between gestational age and the waiting period, and between the number of assisted fertilization attempts with the fear of infertility; it seems interesting to underline that as pregnancy weeks increase, the level of maternal–infant attachment decreases. Data confirm that perinatal loss represents a traumatic process for the parental couple and above all for the mother, who could manifest elevated anxiety during a subsequent pregnancy, thus influencing the quality of the bond with the child [50].

In confirmation of the second hypothesis, in ART pregnant women levels of maternal–infant attachment appear lower, even if the levels of state anxiety appear significantly higher than in women who conceive naturally.

A pregnancy achieved through ART indicates the end of a period of childlessness, which involves several medical treatments. Thus, one could speculate that ART pregnant women would demonstrate higher levels of anxiety given the long periods of infertility they have experienced. This could lead to a reduction in their emotional and affective investment in the pregnancy, as a defensive strategy against the possible frustration resulting from a further failed procreation attempt. In fact, helplessness and the feeling of lack of control over one’s own life could determine an increase in anxiety and dysfunctional coping strategies.

The number of ART attempts seems to be relevant for perceived anxiety, which tends to increase after experiencing several failed attempts [51,52].

In confirmation of the third hypothesis, in the experimental group, the tendency to identify the personality characteristics already attributable to the fetus on the basis of movements (“Differentiation from Self” sub-dimension) increases as the interaction with the fetus increases. In the same way, when the woman’s pleasure at the thought of “another from herself” in her womb increases her ability to imagine herself as a mother in the future (“Role Taking” sub-dimension) also increases.

It appears interesting how the reduced tendency to interact with the fetus and to attribute intentional and personality characteristics to him are correlated to an increase in the maternal–infant attachment. This data confirms the literature that underlines how women with ART appear less interested in documenting the birth of the child, communicate little with the unborn child, and delay all preparations for the birth of the child [29,30].

Maternal–fetal attachment also seems to be negatively correlated with the state anxiety level, since the presence of anxiety about a failed pregnancy could lead to a reduction in the attachment bond with the fetus.

In reference to the last hypothesis, the maternal–fetal attachment is not a significant predictor of the fear of not being pregnant and the perception of a high-risk pregnancy. However, fear of not being pregnant has been found to have a significant impact on state anxiety. Confirming the hypothesis, the total effect of maternal–fetal attachment on anxiety through the mediating variables (fear of not being pregnant and perception of a high-risk pregnancy) was significant.

Similarly, the direct effect of maternal–fetal attachment on state anxiety levels shows a significant impact. This makes sense because state anxiety refers to the conscious and current experience of anxiety symptoms, which might be influenced by the uneasy bond between the mother and the fetus [53].

Within the ICSI pregnant woman, the results revealed that the MFA is not a significant predictor (negative) of the fear of not being pregnant and the perception of a high-risk pregnancy. The role mediated by the fear of not being pregnant seems to have a significant impact on anxiety. Therefore, data suggest that there is no mediation relationship between maternal–fetus attachment and anxiety level when the fertilization technique ISCI was selected.

## 9. Conclusions

The role of ART in the maternal–fetal relationship is still debated today. In contrast to data in the present research, a part of the literature that compares mothers subjected to in vitro fertilization and mothers who conceive naturally demonstrates how the former show a greater bond of attachment than the latter; this leads us to hypothesize that, in women who have difficulty getting pregnant, the desire for motherhood helps build a strong bond of attachment with the fetus.

Previous studies have argued that women who conceived after ART had great difficulty perceiving themselves as pregnant [54], and thus continued to perceive themselves as different from other pregnant women [55].

A recent study conducted by Gamze Teskereci and colleagues [56] demonstrated that levels of readiness to give birth treated with infertility pregnant women were found to be higher than in women who conceived spontaneously, during the end of pregnancy. ARTs, therefore, were more likely to identify with the role of motherhood, childbirth preparation, and birth control; with this completed, they feel more prepared for delivery, but only toward the end of pregnancy, and they define pregnancy as a dream that becomes real. The same study also showed that levels of pregnancy adjustment increased in ART women, as did maternal self-confidence.

Although the present research studies a little investigated phenomenon, such as the pregnancy adaptation precursors and anxiety levels in mothers who conceive naturally and with assisted fertilization, the present study had some limitations.

As the study was conducted during the COVID-19 pandemic, the group of participants is not large.

Due to the pandemic, women’s adaptation to pregnancy, attachment to their babies, and anxiety levels may have been affected. However, this study did not focus on the effects of the COVID-19 pandemic on women’s adaptation to pregnancy and attachment to their babies.

Another limitation is represented by the absence of an assessment of the personality characteristics of the women participating in the research, although a part of the literature underlines that no differences were found in the measure of personality in ART mothers compared to the general population [55]. Conversely, Hjelmstedt and colleagues [56] demonstrated that certain personality traits could represent a risk factor for impaired prenatal attachment in ART mothers.

Furthermore, it is important to emphasize that the different treatment processes, costs, and success of treatment are quite different and that may limit the interpretation of the research.

Considering the limitations of the research and literature, it would be interesting to carry out longitudinal studies to verify the influence of maternal–fetal attachment on subsequent attachment bonds during infancy, manifested not only in the mother–child dyad but also in the parental couple, above all during the sensitive period of growth [57,58,59].

Starting from the results of this research, it is desirable to suggest the use of functional strategies aimed at increasing maternal–fetal attachment, through greater social support within the national health service. Therefore, considering the altered level of attachment in ART pregnant women, it would be appropriate to support these women during the screening test and ultrasound examinations phase.

## Figures and Tables

**Table 1:** Descriptive statistics on the control group.

	N	Min.	Max.	Mean	Std. Dev.
Gestational age	50	24	37	31.20	3.94
Waiting period	50	0	24	2.86	5.10
Fear of not being pregnant	50	0	1	0.46	0.50
Perception of a high-risk pregnancy	49	0	1	0.20	0.41
Presence of other children	50	0	1	0.38	0.49

Note: Gestational age is defined as the number of weeks between the first day of the mother’s last menstrual cycle and childbirth. Waiting period is definitive as the period, expressed in months, from the first attempt to get pregnant to the beginning of the pregnancy. Variables “Fear of not being pregnant” and “Perception of a high-risk pregnancy” are expressed through a 5-point Likert scale.

**Table 2:** Descriptive statistics on the experimental group.

	N	Min.	Max.	Mean	Std. Devi.
Gestational age	45	23	37	28.56	3.60
Waiting period	45	12	96	26.22	14.48
Fear of not being pregnant	45	0	1	0.84	0.37
Perception of a high-risk pregnancy	45	0	1	0.31	0.47
Assisted fertilization attempts	45	1	6	2.40	1.286
Presence of other children	45	0	1	0.07	0.252

Note: Gestational age is defined as the number of weeks between the first day of the mother’s last menstrual cycle and childbirth. Waiting period is definitive as the period, expressed in months, from the first attempt to get pregnant to the beginning of the pregnancy. Variables “Fear of not being pregnant” and “Perception of a high-risk pregnancy” are expressed through a 5-point Likert scale.

**Table 3:** Correlation analysis between independent variable and MFAS in the control group.

Variables	A.	B.	C.	D.	E.	F.
A. Gestational age	-					
B. Age	0.15	-				
C. Waiting period	0.06	−0.04	-			
D. Fear of not being pregnant	0.31 *	0.18	0.06	-		
E. Perception of a high-risk pregnancy	0.65 **	0.18	0.04	0.34 *	-	
F. Presence of other children	0.50 **	0.31 *	−0.04	0.02	0.35 *	-
G. Total MFAS	0.05	−0.24	−0.19	−0.01	−0.06	0.11

Note: ** = *p* < 0.01; * = *p* < 0.05. Note: MFAS is formed by the following sub-dimensions: role taking; differentiation from self; interaction with fetus; attributing characteristics and intentions; and giving of self.

**Table 4:** Correlation analysis between independent variable and MFAS in the experimental group.

Variables	A.	B.	C.	D.	E.	F.	G.	H.
A. Gestational age	-							
B. Age	−0.13	-						
C. Waiting period	0.512 *	−0.08	-					
D. Fear of not being pregnant	0.20	0.29	0.02	-				
E. Perception of the high-risk pregnancy	0.07	0.10	−0.21	0.29	-			
F. Fertilization technique	0.15	−0.22	0.20	−0.15	−0.13	-		
G. Assisted fertilization attempts	0.26	−0.12	0.10	0.33 *	−0.02	0.05	-	
H. Presence of other children	0.34 *	−0.02	0.56 **	0.12	−0.18	0.22	0.13	-
I. Total MFAS	−0.32 *	−0.09	0.07	−0.16	−0.08	0.28	0.02	0.03

Note: ** = *p* < 0.01; * = *p* < 0.05. Note: MFAS is formed by the following sub-dimensions: role taking; differentiation from self; interaction with fetus; attributing characteristics and intentions; and giving of self.

**Table 5:** The Independent Samples T Test: MFAS and STAI comparing the experimental and control group.

Dimensions	Sub-Dimensions	Group	N	M.	S.D	T	*p*-Value
MFAS	Role Taking	Experimental Group	45	17.00	2.11	0.76	
		Control Group	50	16.68	2.00	0.76	0.45
	Differentiation from Self	Experimental Group	45	15.91	2.10	0.94	
		Control Group	50	15.56	1.54	0.92	0.36
	Interaction with the fetus	Experimental Group	45	17.00	2.41	−0.42	
		Control Group	50	17.20	2.24	−0.42	0.68
	Attributing characteristics and intentions	Experimental Group	45	20.80	3.18	1.43	
		Control Group	50	19.96	2.55	1.41	0.16
	Giving of Self	Experimental Group	45	18.76	1.84	0.86	
		Control Group	50	18.34	2.73	0.88	0.38
	Total MFAS	Experimental Group	45	100.87	17.90	−4.44	
		Control Group	50	115.01	12.98	−4.37	0.00
STAI	STAI S (State anxiety)	Experimental Group	45	43.27	9.62	2.06	
		Control Group	50	39.28	9.24	2.06	0.04
	STAI T (Trait anxiety)	Experimental Group	45	44.47	7.55	1.59	
		Control Group	50	41.94	7.92	1.59	0.12

**Table 6:** Correlation analysis between MFAS and STAI in the experimental group.

Variables	A.	B.	C.	D.	E.	F.	G.
A. Role taking	-						
B. Differentiation from self	0.37 *	-					
C. Interaction with fetus	0.33 *	−0.04	-				
D. Attributing characteristics and intentions	0.21	−0.07	0.26	-			
E. Giving of self	−0.14	−0.25	0.27	0.13	-		
F. Total MFAS	−0.27	0.16	−0.37 *	−0.53 **	−0.17	-	
G. STAI S	0.13	0.23	0.12	0.26	0.01	−0.38 **	-
H. STAI T	−0.04	0.09	0.05	0.28	−0.21	−0.44 **	0.46 **

Note: ** = *p* < 0.01; * = *p* < 0.05. Note: MFAS is formed by the following sub-dimensions: role taking; differentiation from self; interaction with fetus; attributing characteristics and intentions; and giving of self.

**Table 7:** Mediation effects summary.

Total Effect IV- > DV	Direct Effect IV- > DV	Relationship	Indirect Effect	Confidence Interval		t-Student	Conclusion
				Lower Bound Upper Bound			
−0.206 (0.009)	−0.211 (0.005)	IV Hypothesis IV- > MV1-VD Hp 4VI > MV2- > V	0.0060; SE = 0.0331	−0.0592	0.0769	−2.720	Partial mediation

Abbreviations: IV = independent variable; DV = dependent variable; MV1 = fear of not being pregnant; MV2 = perception of the high-risk pregnancy; SE = standard error.

## Data Availability

Data supporting the conclusions of this article will be made available by the authors, without undue reservation.
